# Tropical forest disturbances reveal increase in stress-tolerant(s) strategy among epiphytes while simplifying the taxonomic and layer structure of epiphytic communities

**DOI:** 10.3389/fpls.2026.1695534

**Published:** 2026-02-18

**Authors:** Alen K. Eskov, Evgenia A. Faronova, Tatiana G. Elumeeva, Taisia V. Poloshevets, Anna S. Kartasheva, Nikolay G. Prilepsky, Vlad. D. Leonov, Evgeny V. Abakumov

**Affiliations:** 1 Department of Plant Ecology and Geography, Moscow State University, Moscow, Russia; 2 Polar-Alpine Botanical Garden-Institute of Kola Scientific Centre of the Russian Academy of Sciences, Apatity, Murmansk, Russia; 3 Department of Botany, State Museum of Natural History, Stuttgart, Germany; 4 Severtsov Institute of Ecology and Evolution of the Russian Academy of Sciences, Moscow, Russia; 5 Department of Applied Ecology, Saint-Petersburg State University, St. Petersburg, Russia

**Keywords:** CSR strategies, epiphytes, functional traits leaf, Grime’s theory, tropical forest disturbances, Vietnam

## Abstract

One of the most popular approaches in functional plant ecology is the study of CSR strategies based on Grime’s theory. However, this approach to the study of epiphytes has not been used yet. We assumed that the response of epiphytes to disturbances would be different than that of terrestrial plants. Namely, this would lead to a decrease in epiphytes with the competitive (C) strategy and an increase in the number of stress-tolerants (S) in disturbed forests. We found that in primary forests, representatives of the Orchidaceae family dominate in terms of species number, while in disturbed forests, Orchidaceae and Polypodiaceae dominate. Epiphytes demonstrate a tendency to a more pronounced C- strategy than tropical forest trees and to a more R- strategy than terrestrial herbs. At the same time, most epiphytes gravitate toward the radical S- strategy. In the primary forest, epiphytes adhering to competitive, ruderal, and mixed strategies are widely represented. Representatives of these strategies disappear in secondary forests so that predominantly (S) stress-tolerant and one (C) competitive species remain. In the studied secondary formations of tropical forest, the lower forest layer is occupied by succulent orchids and ferns. Undisturbed tropical forest is characterized by the presence of sciophytic and mid-stem epiphytes. Disturbance of the tropical forest structure leads to the loss of epiphytic species of the lower synusiae, while the advantage passes to stress-tolerant succulents. Thus, the change in the functional diversity of epiphytes is directly related to the change in the structure and layering of the forest canopy

## 1 Introduction

Epiphytes exist in the forest canopy, they are structurally dependent plants, thus the patterns of their dispersal may differ from normal terrestrial vegetation (Eskov and Kolomeitseva, 2022). Epiphytes disperse in a three-dimensional environment and depend on both the phorophyte and the characteristics of this environment - the forest canopy, which, in turn, depend on the topography and climatic conditions (Watkins et al., 2006; Hietz, 1997). The tropical forest is a complex, multi-tiered structure that provides numerous diverse habitats for epiphytes. Epiphytic communities continuously colonize the substrate unsuitable for plant life. They disperse т unevenly, often individuals of the same species grow in clusters on the same tree - because not all potential phorophytes have suitable characteristics: texture and porosity of the bark, thickness of branches and trunk. Many studies pay attention to the confinement of epiphytes to certain parts of the phorophyte; there are different tree zoning schemes - since on the edges of the crowns, in the center of the trunk and at its base, the conditions of moisture availability, insolation conditions and mechanical characteristics of the support differ significantly (Johansson, 1974; Benzing, 1981; Scipioni et al., 2022; Shaw, 2004). It should be emphasized that different approaches can be used to study the structure of the epiphytic community: epiphytes can be taken into account on the site (as part of the phytocoenosis) (Mendieta-Leiva and Zotz, 2015; Linares-Palomino et al., 2009), within the tree (as a consortium of a specific phorophyte or phorophyte species) (Cascante-Marín et al., 2006; Quaresma and Jardim, 2012; Boelter et al., 2014) or vertical zones of the tree (as epiphytic synusia) (Irume et al., 2013; Mehltreter et al., 2005). On the other hand, there is no single methodology for describing epiphytic communities, which makes it problematic to compare the results of different studies, although attempts at standardization are being made (Zotz, 2016; Mendieta-Leiva and Zotz, 2015).

In natural conditions, the death of epiphytes is mainly associated with two factors: lack of moisture and dropping down from a height. Mechanical damage caused by both biotic and abiotic factors affects epiphytes quite differently than terrestrial plants (Cabral et al., 2015; Gomez-Escamilla et al., 2017). Dropping bark can easily tear off small epiphytes, while larger individuals are more securely attached and are easily held. On the other hand, branches break under both large and small individuals, but branches with many epiphytes attached break more easily. Large specimens often die due to substrate instability, while small ones die for various reasons, but most often due to drying out (Hietz, 1997; Zotz, 1998; Zotz et al., 2005, 2021).

It has been noted that in forests that have been subjected to anthropogenic disturbances without complete destruction of the stand, drought-tolerant species begin to fall out, since juvenile individuals of epiphytes are more vulnerable to changing microclimatic conditions (Parra Sanchez and Banks-Leite, 2022). At the same time, in secondary stands, the number of individuals and species diversity of epiphytes decrease, compared to primary forests (Eskov et al., 2020). Thus, in disturbed tropical forests, the complexity of the epiphytic community decreases, since the habitat itself changes. Aforestation or degradation of the primary tropical forest leads to the disappearance of epiphytes growing in the lower part of the trunk, in the shade, which cannot disperse into agroecosystems s or secondary formations and thus directly depends on the layering and complexity of the forests of growth (Eskov, 2013). This is due to serious microclimatic changes under the canopy during its degradation. Thus, in the flat undisturbed high-trunk tropical forest of South Vietnam, the illumination at the base of the trunks is 200–400 lx, and the humidity is 95-98%, while in the crowns of the first-tier trees the illumination reaches 150,000 lx, with humidity of 75-80% and significant daily humidity fluctuations (Kuznetsov, 2003).

This dependence of epiphytic communities on microecological conditions at the scale of the stem layer suggests the relevance of studying the functional traits of epiphytic plants, as reflecting the relationship between the biological and ecological characteristics of plants, their adaptability to environmental conditions (Pérez-Harguindeguy et al., 2013). Functional traits are measurable quantitative objective parameters to which statistical methods are applicable, which allows us to compare geographically distant communities, as well as communities with different taxonomic compositions (Korablyov and Smirnov, 2017). Thus, the study of functional traits is promising for the study of epiphytic communities, since it can solve the problem of methods that cannot be compared. In epiphytes, the functional traits of the leaf are best studied, since they are easy to measure in the field, while data on the functional traits of roots and diaspores are insufficient (Zotz et al., 2021). Epiphytes do not typically survive during unfavorable period of the year in the form of seeds, since They do not have time to fulfil their life cycle in one season. The majority of epiphytes are evergreen plants, with the exception of some orchids with succulent photosynthetic pseudobulbs and seasonally shed thin leaves (e.g. *Lycaste, Cyrtopodium, Acineta*). The limited occurrence of deciduousness in epiphytes is associated with the need to save mineral nutrition elements - by analogy with terrestrial plants living on poor substrates (Zotz, 2016). Thus, epiphytes are practically deprived of the opportunity to use adaptations to unfavorable periods during the year (dry season), which are common in trees and terrestrial grasses (Suriyagoda et al., 2018; Vergutz et al., 2012; Zotz et al., 2021). The functional characteristics of epiphytes differ from the functional characteristics of woody and terrestrial herbaceous plants. Based on the global dataset, it was found that epiphytes differ from terrestrial on soil above ground plants by their thicker leaves and higher water content in the leaf. The leaf area of an epiphyte can be comparable to that of a woody plant, but the specific leaf surface is larger in terrestrial herbs. That is, the leaves of epiphytes are more succulent (Hietz et al., 2021).

Large taxonomic groups of epiphytes also have significant differences in functional traits: this is associated with phylogenetic adaptations of these groups, which are pronounced even if a particular species has an atypical strategy. In particular, the water content and leaf blade thickness are greater in orchids than in ferns. Within one taxonomic group, differences between terrestrial and epiphytic representatives are also significant. As established by Helsen et al. (2023), comparing 47 terrestrial and 34 epiphytic fern species, epiphytic species have, on average, a smaller leaf area and specific leaf area, as well as a greater equivalent water thickness (succulence) than terrestrial species. A significant role of lack of moisture and mineral nutrition elements in the formation of epiphyte traits is noted, but environmental gradients also play a significant role. Vertical gradients of insolation, moisture, and nutrient availability along the stem can be greater than horizontal gradients within the landscape (Johansson, 1974). This means that when conditions change, epiphytes can more easily migrate to other parts of the stem than disperse horizontally like terrestrial plants (Guzmán-Jacob et al., 2022). However, data on the relationship between morphological and functional leaf traits in epiphytes across stem parts – as well as across landscapes – are ambiguous. Variations in leaf morphological traits within spatial and ecological scales are difficult to disentangle (Messier et al., 2010), partly because “soft” (i.e. easily measured) traits are indicative of mechanically related characteristics to plant performance (Wright et al., 2010). Some epiphytic species grow throughout their life and therefore leaf morphological features will differ among individuals of different ages – this has been shown for the Bromeliad family, which is absent from the Old World. For example, the specific leaf area (SLA) of the tank bromeliad *Vriesea sanguinolenta* differed between small and large ones by more than four times (Zotz et al., 2004). In the long term, this may complicate comparisons among regions, since it reduces the significance of “species features” to zero (Schmidt and Zotz, 2001; Zotz and Asshoff, 2010). Measurement of leaf dry matter content (LDMC) of epiphytes in the Kilimanjaro mountain forests showed that LDMC of epiphytes increases moderately with altitude, while terrestrial plants show a significant increase in water content with altitude (1400 m gradient) (Schellenberger-Costa et al., 2018). The authors interpret this as a response to temperature changes, since the air humidity level did not change. However, some studies show that functional traits (SLA) respond somewhat differently to landscape changes (shaded coffee plantations or tropical forest) and to the vertical gradient of trees (Richards and Damschen, 2022). Obviously, the problem of studying the morphological functional traits of vascular epiphytes requires further study, since there is not enough research on this topic: almost all the researchers mentioned above note that in order to study the functional traits of epiphytes, it is necessary to adapt methods for terrestrial plants.

One of the most popular approaches in functional plant ecology is currently the study of CSR strategies based on the theory of Grime (1988), according to which plants have different strategies for adaptation to two main factors: stress and disturbances. Under conditions of high stress and strong disturbances, plants cannot exist; under conditions of strong stress and weak mechanical disturbances, stress-tolerates (S) gain an advantage; under weak stress and strong mechanical disturbances, ruderals (R) gain an advantage, and in the absence of both, competitive species (C) are the most effective. For a long time, the classification of plants into different types of strategies was based on the analysis of numerous data, but in recent decades a number of studies have appeared that make it possible to quantitatively assess the contribution of different strategies, including based on the analysis of a few functional traits of the leaf (Pierce et al., 2013; Verheijen et al., 2016). The most popular method here is based on the comparison of three leaf traits: dry and water-saturated mass and leaf blade area (Pierce et al., 2017). These three primary traits are very important for plant functioning; they also allow calculating important derived traits, such as specific leaf area (SLA) or leaf dry matter content (LDMC). Functional traits allow linking the characteristics of plant leaves with its CSR strategy. Stress-tolerant plants have small leaves with high water content, ruderal plants have low dry matter content and high water content, and competitive species are distinguished by a large leaf area (Pierce et al., 2013).

However, studies of the morphological functional traits of epiphytes in Indochina and Vietnam in particular have not been conducted, as well as the study of CSR strategies of epiphytes in general. Therefore, the aim of this work is to study how the CSR strategies of epiphytes in tropical ecosystems changed under significant anthropogenic impacts. We were guided by the following hypotheses: (1) the taxonomic composition of epiphytic communities will be simplified in disturbed communities, which is associated with the simplification of the forest structure, an increase in moisture deficit stress and an increase in insolation in the lower part of the trunk and, in turn, this (2) will lead to a decrease in epiphytes with a competitive strategy (C) and an increase in the number of stress-tolerants (S) in disturbed forests.

## 2 Materials and methods

### 2.1 Study area

**Figure 1:**
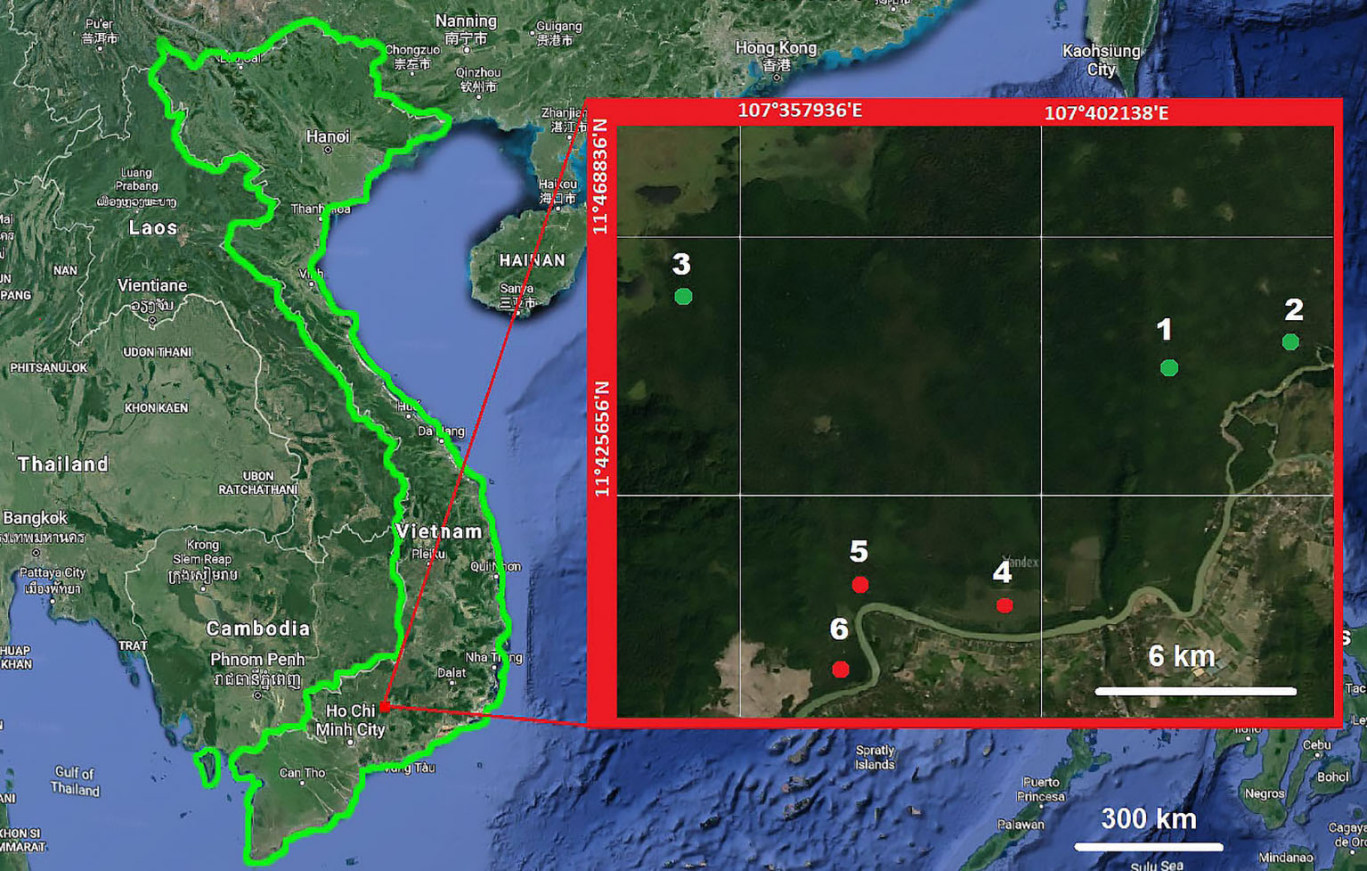
The study area: a satellite map Cat Thien National Park (Nam Cat Tien Sector) with six sampling sites marked by colored circles, against the background of the general map of the region. Areas in the primary forest are marked in green, disturbed areas in red. The following area are numbered: (1) Mixed High Forest (MHF), (2) Lagerstroemia Forest (LF), (3) Crooked Low Forest (CLF), (4) Linear Plantings (LP), (5) Forest Plantation (FP), (6) Forest Plantation clear (FPC).

The material for the study was collected during expeditionary work in Vietnam in May-June 2023. The study of epiphytic communities was conducted in Cat Tien National Park, which is located in Dong Nai Province, approximately 150 km northeast of Ho Chi Minh City, at the foot of the Central Plateau of Vietnam (**Figure 1**). Since 2011, Cat Tien, together with the Vinh Cuu Nature and Cultural Reserve, has been included in the Dong Nai Biosphere Reserve. Cat Tien National Park was founded in 1978. The territory of the national park occupies approximately 74,000 hectares; and is divided into 3 sectors: Cat Loc, Tay Cat Tien and Nam Cat Tien, the largest (38 thousand hectares), in which the research was conducted.

In the Nam Cat Tien sector under study, the relief is flat and gently undulating, with an elevation difference of 170–150 m. The underlying parent materials are composed of shales overlain by basalts (Fromaget, 1971); these metamorphic and igneous deposits were formed in the late Pleistocene as a result of the activity of the ancient shield volcano Dong Nai Thuong (Smithsonian institute.com). The sector under study is located on the right bank of the Dong Nai River, in its middle reaches. The river water level depends on the season, and during the flood the water level rises by 3–4 m; the width of the river is 20–60 m (Nguyen Van Thinh and Anichkin, 2011). During the wet season, numerous tributaries of the Dong Nai are filled with water, and the swamp-lake areas are combined into a single hydrological system (Deshcherevskaya et al., 2013). Climatic and edaphic parameters are presented in **Table 1**.

**Table 1:** Edaphic and climatic conditions of the habitats studied.

Location	Nam Cat Tien Sector, Cat Tien National Park, Dong Nai Province
Mean annual precipitation	2470mm
Dry season	November – April
Mean annual temperature	26.2°C
Soil	Dystric Skeletic Rhodic Cambisols and Skeletic Greyzemic Umbrisols

Climatic features are given according to Deshcherevskaya et al., 2013, soil features according to USS Working Group WRB. World Reference Base for Soil Resources, 2022.

### 2.2 Flora and vegetation

The zonal vegetation type for the lowland areas of Southern Vietnam and, in particular, for Cat Tien National Park, are tropical monsoon semi-deciduous forests. The forests of Cat Tien have been described in details in a numerous of publications (Blanc et al., 2001; Kuznetsov and Kuznetsova, 2011; Eskov et al., 2020). In the 20th century, the forests of Cat Tien were subjected to significant anthropogenic changes during the 1st and 2nd Indochina Wars. The forest served as a refuge for the Viet Minh and Viet Cong forces; military zone D was located on the territory of the future national park (Kuznetsov et al., 2010). The ecological war implemented a strategy of destruction of vegetation in order to make it impossible for enemy forces to exist in this territory and advance through it. As a result of intensive military actions, part of the massif suffered from bomb explosions, napalm and defoliants. The species that form the upper tier of tropical forests in the territory of Cat Tien turned out to be the most sensitive to the effects of defoliants: *Dipterocarpus dyeri* Pierre, *D. obtusifolius* Teijsm. ex Miq., *Shorea cochinchinensis* Pierre, *Sindora cochinchinensis* Baill (Phung Tuu et al., 1994). Nevertheless, despite the defoliant treatments, some tropical forests were able to preserve their structure. In place of forests destroyed by napalm and defoliants, after the upper soil horizons were washed away, stable grasslands formed, which are not typical for the landscapes of Southern Vietnam, with the dominance of tall representatives of the Poaceae family (*Imperata cylindrica* (L.) Raeusch., *Pennisetum polystachion* (L.) Schult., *Themeda arundinacea* (Roxb.) A.Camus) (Kuznetsov et al., 2010). These grasslands, taller than a human, are partly present on the territory of the National Park and are not subject to overgrowth, thus becoming a stable ecosystem. In addition, valuable tree species were cut down here, which was stopped only by 1990 (Kuznetsov et al., 2002). Later, since 1995, by decree of the Vietnamese government and later, with the support of the European Commission, a forest cover restoration program was carried out here (Millet, 2006). Thus, the forests of Cat Tien represent a unique study site, as they contain both areas of primary tropical forest and areas where the forest cover has been cleared and then replanted.

### 2.3 Field research sites

We studied 6 sites: 3 in primary mature forests and 3 in secondary communities (**Figure 1**). The sites were identical in topography - located on non-flooded flat areas; different in the number of tiers and, presumably, in microclimatic conditions for epiphytes. In this study, we considered primary forest sites to be those in which adult trees of the first tier, the height of which reaches 40–50 m, and the entire forest thickness under them have been preserved, which allows us to assume the preservation of microclimatic features inherent in tall-stemmed tropical forests: high air humidity and lower, relative to disturbed and deforested areas, heating of the surface air layer (22-28˚C during the day) (Kuznetsov, 2003). The selected sites differ in the composition of the tree stand. We studied the following communities: (1) Mixed high forests (MHF) – located in a forest that is virtually untouched by logging, north of the national park estate, in the vicinity of the so-called Circular Trail – the most frequently visited by researchers. The upper tier is dominated by *Lagerstroemia calyculata*, *Sterculia* sp., *Terminalia calamansanai* (Blanco) Rolfe, *Tetrameles* sp., *Afzelia xylocarpa*, *Ficus* sp. The second tier, 20–25 m high, is dominated by trees of the genera *Dalbergia, Knema, Acronychia*. The third tier, 2–5 m high, is dominated by *Randia, Ardisia, Grewia*, and occasionally cycads (*Cycas inermis* Lour.) and palms (*Licuala*). The herbaceous tier is poorly developed, with a TPP of 5%. (2) Lagerstroemia forest (LF) – in this area, the upper tier was dominated exclusively by *Lagerstroemia* spp. In the second tier, there are also many growing Lagerstroemias, Dipterocarpaceae and Fabaceae are noted, the height of the trees of the second tier here is on average 25 m. The third tree tier and the herbaceous tier are similar to those noted earlier, in a mixed forest (*Psychotria, Livistona* and other palms). (3) Crooked low forest (CLF) – the third site was in a low undisturbed forest located on a gentle slope of a hill with a north-western exposure, on the path to Lake Bau Sau. This site is distinguished by the characteristic dominance of *Randia* sp. in the lower tier, 4–6 m high, with a density of 0.3-0.6. Secondary – these are forest areas in which the primary tree stand has been completely destroyed. In place of the forest, tall grasslands were formed with dominance of sedges and cereals and with isolated Fabaceae trees. Later, in 1996-1998, on the deforested territory to the east of the village of Talay, forest plantations imitating primary forest (the so-called “French plantations”) were planted with dominance of Dipterocarpaceae (*Dipterocarpus alatus, D. dyeri, Hopea odorata* Roxb., *Anisoptera costata* Korth.); 6 species of Lythraceae from the genus Lagerstroemia; 7 species of Fabaceae belonging to the genera *Afzelia, Sindora, Dalbergia, Pterocarpus* (Millet, 2006). It was assumed that plantation management would consist of weeding of large grasses in the understory to reduce the risk of fire, however, to date, management includes clearing of emerging understory and vines, in particular *Calamus* sp. Both 4) Forest plantation (FP) and (5) Forest plantation clear (FPC) were studied. The height of the overstory of the forest stand in the uncleared plantations was 25 m, while in the cleared plantations it was on average 30 m. In addition to the forest plantation areas, (6) Linear plantings (LP) a wide shelterbelt planted in grasslands along the road to Ta Lai village, in the vicinity of Nuy Tuong cordon, were studied. The trees of the shelterbelt are also dominated by *Dipterocarpus* spp. and *Lagerstroemia* sp*eciosa* (L.) Pers. The height of the forest stand in the forest belt reached 25 m. In all disturbed communities, a high area of projective cover of the herbaceous layer (50-70%) is observed. Among the herbs, representatives of the families Zingiberaceae (*Globba* sp., *Alpinia* sp.); Poaceae (*Imperata cylindrica, Paspalum* sp.) and Cyperaceae (*Kyllinga* sp.) dominate; in the plantations, the ground vegetation cover is clearly divided into two sublayers, with ginger, composites and wild sugar cane in the upper tier, 1–2 m high, and with sedges, cereals and polypodial ferns 20–50 cm high.

### 2.4 Field research

Three sample plots were laid out in each community. The sample plots size was 50*50 m; in linear forest plantations 10*250 m. The sites were selected subjectively, based on the visual perception of their species richness (since the sites with the highest species richness were selected) The methodology for compiling the geobotanical description was adapted to the conditions of the tropical forest and the objectives of the study (Yunatov, 1964; Richards, 1961). When compiling the descriptions, the main task was to display the nature of the vegetation cover, taking into account the abundance of the most common species as a percentage of the projective cover area. Quantitative accounting of epiphytic vegetation was carried out by visual observation, samples were collected using a telescopic pole system (Eskov et al., 2020). Taking into account height and partial tree climbing (up to large forks without the use of special equipment), the maximum height reach was approximately 30 m. When counting, the tiered confinement of epiphytes was taken into account: heliophilic - on the peripheral parts of the crown or on skeletal branches, but spending at least part of the day in direct light; mid-trunk - living in the middle part of the trunk and receiving direct lighting only in the form of a light mosaic; sciophytic - in the coarse part of the trunks, on plank roots or on small phorophytes of the 3rd tier, receiving light only in the form of diffused lighting. We classified the epiphyte into the main biomorphological groups such as succulent or accumulative epiphytes based on our own data on the presence of CAM (in general, for a number of national parks in the region, we described data on the 13C isotope, which is a good diagnostic of the type of photosynthesis and succulence - Eskov et al., 2023) and the formation of mechanisms for the accumulation of suspended soils in accumulative epiphytes, for example, in many ferns from the Polypodiaceae (the mechanisms and features of this process have been described in detail by us – Abakumov and Eskov, 2023; Eskov et al., 2025). Succulent epiphytes in this classification are, as a rule, species with CAM and xeromorphosis of axial organs (to varying degrees), and accumulative ones are large plants (usually with C3 metabolism) that form certain organs for retaining litter, forming suspended soils, etc. Species were determined in cameral conditions, using the Flora of Vietnam (Hô, 2000).

### 2.5 Measurement of functional traits

To measure the functional traits, intact, fully expanded leaves of plants were selected in 5 replicates in each community. In each key plot, leaves were collected from all recorded epiphyte species; in addition, leaves from the 10 most common woody plant species and 10 most common herbaceous species were collected. Thus, from each type of epiphyte, from 5 (if it was found on one site) to 30 leaves (if it was found on all sites) were examined. In laboratory conditions, measurements of leaf mass in a water-saturated state, leaf cross-section and area, leaf mass in an air-dry state were carried out, as well as calculations of the specific area of a water-saturated leaf, the specific area of a dry leaf, and the percentage of water in the leaf. Leaves in a water-saturated state were dried with a paper towel and weighed on an analytical balance (accuracy up to 0.1 mg) to obtain the leaf mass in a water-saturated state (LFM). The weighed leaves were then placed on a sheet of paper and scanned at a resolution of 300 dpi for medium- and large-sized leaves and 600 dpi for small leaves with a midrib length of less than 1.5 cm. The scanned leaves were placed in paper envelopes and dried for at least 24 hours in a drying cabinet at 80 °C. Each leaf was then weighed again to obtain the leaf dry mass (LDM).

To calculate the area, the leaf area (LA) was estimated in pixels and then converted to dm ^2^ using the formula:


$$\text{LA }={\text{N}}_{\text{pixels}}\text{Х}2.54^{2}/\text{DP}{\text{I}}^{2},$$

where LA is the desired area, N_pixels_ is the number of pixels in the leaf image (calculated by a raster editor: ImageJ, Adobe Photoshop, GIMP), 2.54 is the size of one inch in centimeters, DPI is the scanning resolution (dots per inch) (Pierce et al., 2017; Soudzilovskaia et al., 2013).

Based on the data on the mass of the watered and dried leaf, as well as the leaf area, other functional characteristics were calculated:


$$\text{LSI }\left(\text{leaf succulence index}\right)\;=\;\left(\text{LFM }-\text{ LDM}\right)/\left(\text{LA}/10\right),\text{ g water d}{\text{m}}^{-2}$$

LDMC (dry matter content) = (LDM/LFM) x 100, %

$$\text{SLA }\left(\text{specific leaf area}\right)\;=\text{ LA}/\text{LDM},\text{ m}{\text{m}}^{2}\text{m}{\text{g}}^{-1}$$

An electronic micrometer was used to measure the leaf cross-section thickness (LTH). The leaf was measured in the area of the central vein with an accuracy of fractions of a mm. A total of 754 leaf samples were processed, of which 370 were samples of leaves of epiphytic plants.

### 2.6 Statistical processing

Using the StrateFy calculator (Pierce et al., 2017), the computable functional traits (LSI, LDMC and SLA) were calculated, as well as the CSR strategy for each epiphytic species encountered in the formation, as well as for mass woody and herbaceous. The ggtern package (Hamilton and Ferry, 2018) was used for visualization of ternary CSR strategies with two-dimensional probability density distributions of strategies scores, derived through kernel density estimations. To compare leaf functional traits and strategy scores of epiphytes between communities we used k-sample Fisher-Pitman permutation tests with Monte-Carlo approximation and pairwise multiply comparisons with a false discovery rate control in R packages “coin” and “rcompanion” (Hothorn et al., 2008; Mangiafico 2022). The permutation tests were used, because the data did not comply the normality assumptions and have different number of species in every community. We applied the same analysis to compare the trait values and strategy scores between epiphytes, woody plants and terrestrial herbs within every community.

## 3 Results

### 3.1 Description of the epiphytic community

**Figure 2:**
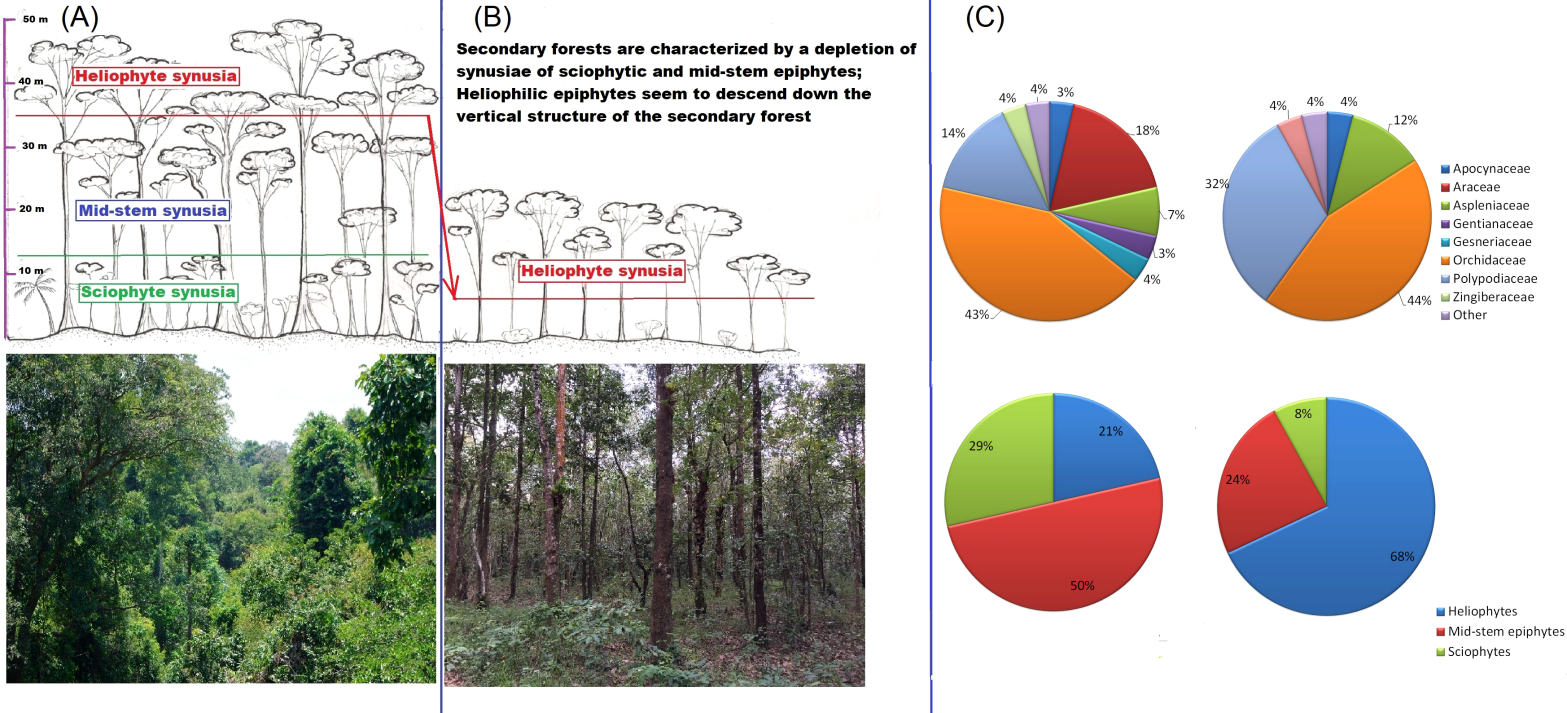
The vertical structure of primary **(A)** and secondary **(B)** forests: the profile diagram shows schematically that the main changes in the transition from primary to secondary forests occur due to the simplification of the vertical structure, as a result of which heliophyte epiphytes seem to “descend”. The insets below show what the studied forest in Cat Tien looks like: on the left, Mixed high forest (MHF) at an altitude of about 40 m, on the right, Forest plantation clear (FPC) at human height. **(C)** taxonomic (upper row of circular diagrams) and tiered structure (lower row) of epiphytic communities of the primary (left) and secondary (right) studied areas of the Cat Tien forest. The taxonomic structure is indicated by the number of species, and the tiered structure (belonging to one of the three vertical synusia) by the abundance of epiphyte species.

A total of 46 species of epiphytic plants belonging to 8 families were found of which 7 species are common to both primary and secondary communities. In secondary communities, a total of 25 species from 5 families were noted, with representatives of Orchidaceae and Polypodiaceae in approximately equal numbers (**Figure 2**). In primary communities, a total of 28 species from 8 families were noted, with the largest number of species in the Orchidaceae family (**Figure 2C**). Representatives of the Zingiberaceae and Araceae families were noted in undisturbed communities, but never found in disturbed ones (**Figure 2C**). The tier characteristics of the epiphytic communities also differed. In secondary forests, heliophytic epiphytes dominate (in terms of abundance, but not in terms of the number of species) - almost 70%, while in primary forests the proportion of medium-sized epiphytes is greater, almost 50%, but the proportion of sciophytes is also large, almost 30% (**Figure 2C**).

### 3.2 Functional traits of leaves

**Figure 3:**
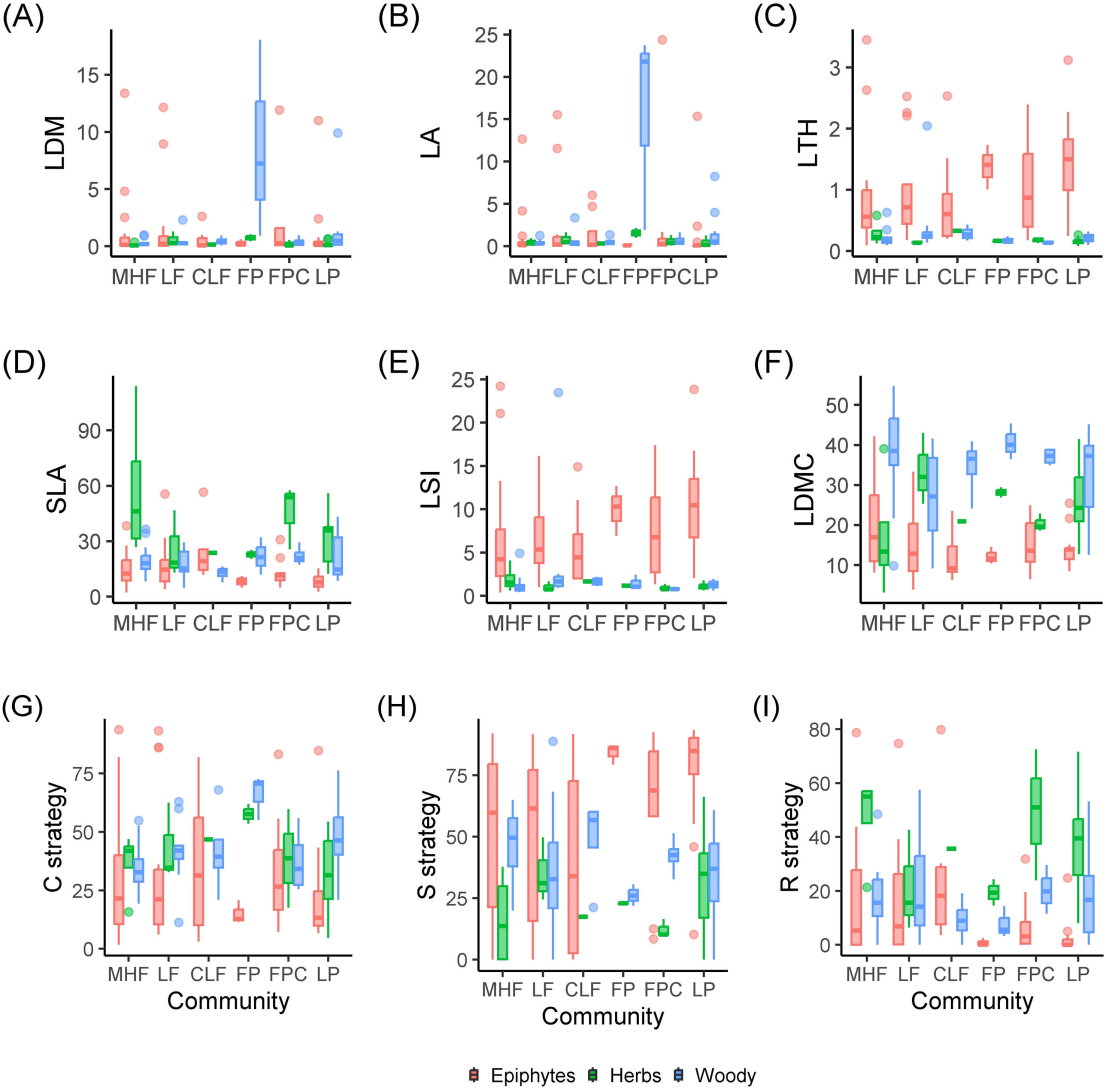
Functional traits **(A–F)** and strategy scores **(G–I)** of epiphytes, woody plants (trees and lianas) and terrestrial herbs in different primary and secondary plant communities. Boxes show 95-% confidence interval, lines are medians and points are outliers. Primary communities: MHF, mixed high forest; LF, Lagerstroemia forest; CLF, crooked low forest. Secondary communities: FP, forest plantation; FPC, forest plantation clear; LP, linear plantings. Functional traits: **(A)** LDM, leaf dry mass, g; **(B)** LA, leaf area, dm^2^; **(C)** LTH, leaf thickness, mm; **(D)** SLA, specific leaf area, mm^2^ mg^−1^; **(E)** LSI, leaf succulence index, g water dm^−2^; **(F)** LDMC, leaf dry matter content, %; **(G)** competitive strategy score, %; **(H)** stress-tolerance score, %; **(I)** ruderal strategy score, %. Significance of differences between traits of epiphytes, trees and terrestrial herbs within communities is given in the **Supplementary Table**.

**Figure 4:**
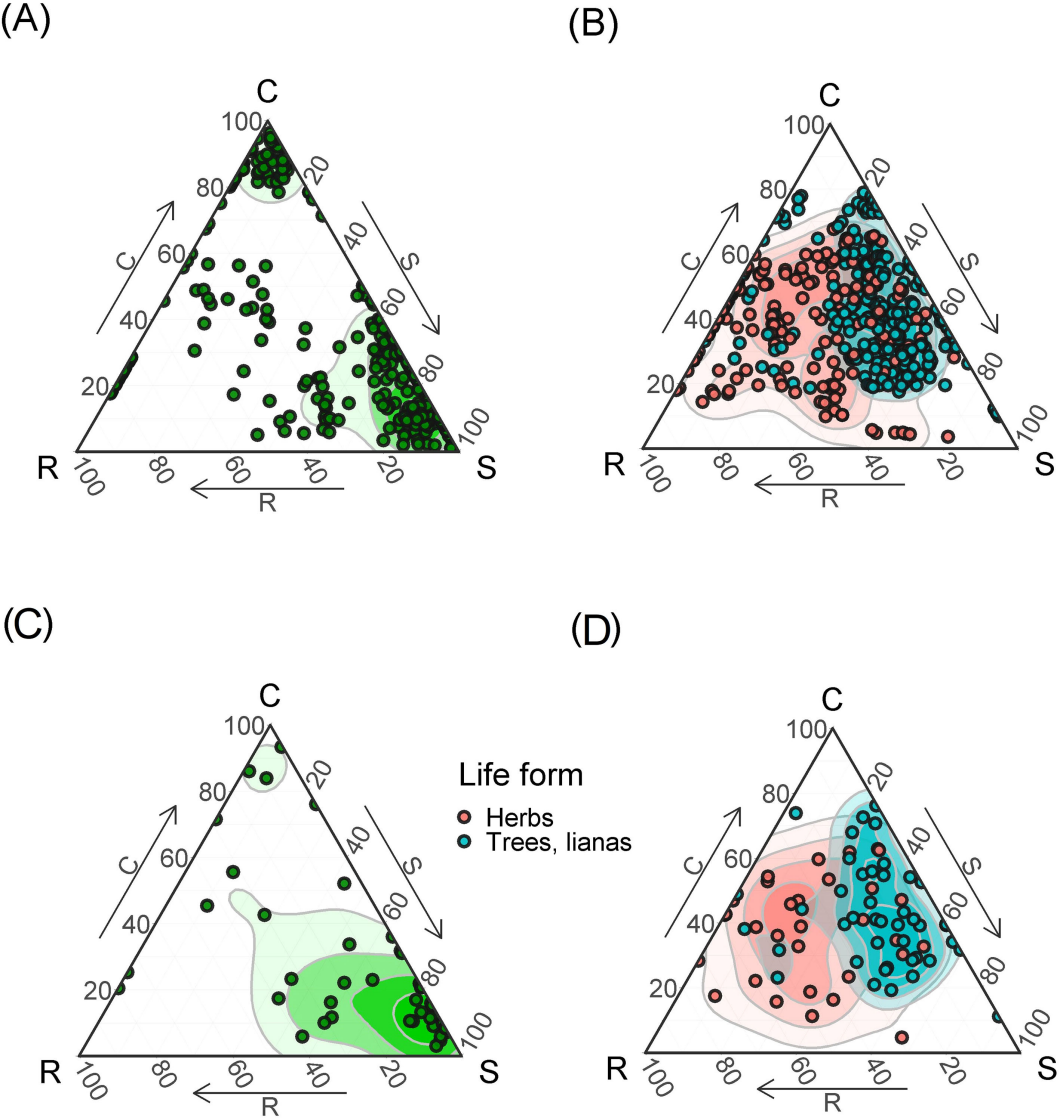
The SCR strategies of epiphytes **(A, С)** and terrestrial plants **(B, D)** woody (trees and lianas) and terrestrial herbs. The points on triangles at the top show each measurement. Points on triangles at the bottom per species means of strategies scores. The points for the species are indicated according to the “species-area” principle, respectively, here and below on common and separate triangles one species can have different views. In some cases, with similar values, the points of the views merge. Contour lines show two-dimensional probability density distributions of strategies scores derived by kernel density estimations. The color gradient indicates regions of highest (dark) and lowest (white) occurrence probability of species with the given strategy.

**Figure 5:**
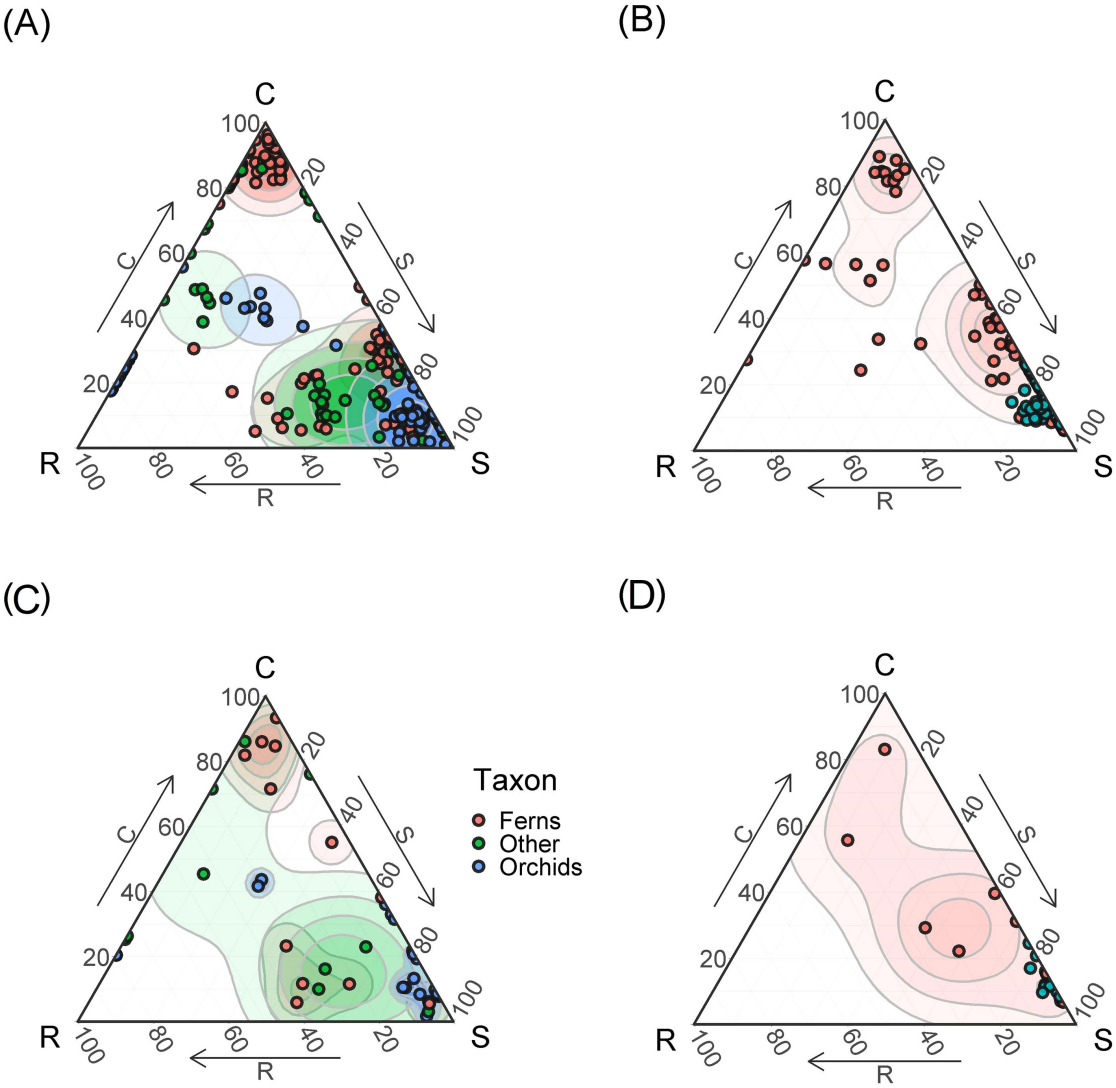
The SCR strategies of epiphytes belonging to different taxon groups (ferns, orchids and others) in primary **(A, С)** and secondary **(B, D)** forests. The points on triangles at the top show each measurement. Points on triangles at the bottom per species means of strategies scores. Contour lines show two-dimensional probability density distributions of strategies scores derived by kernel density estimations. The color gradient indicates regions of highest (dark) and lowest (white) occurrence probability of species with the given strategy.

**Figure 6:**
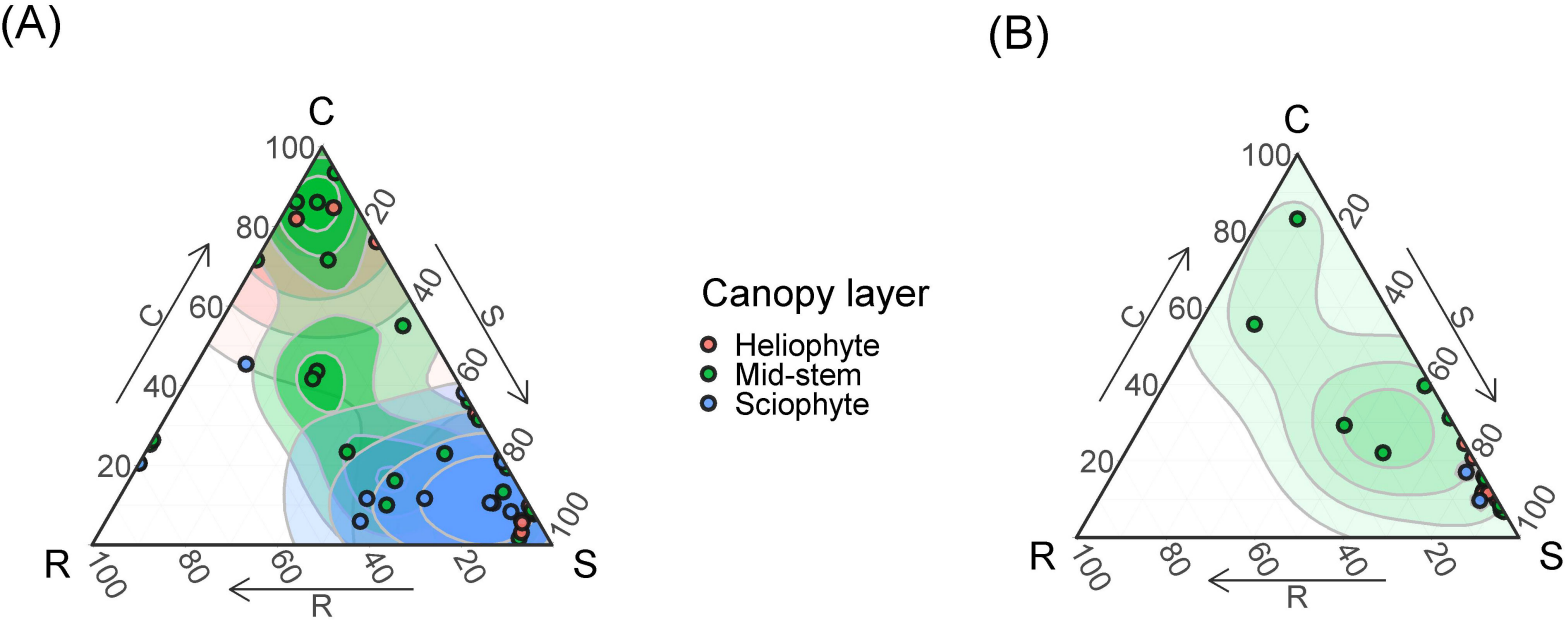
The SCR strategies of epiphytes belonging to different canopy layers (heliophytes, mid-stem epiphytes and sciophytes) in primary **(A)** and secondary **(B)** forests. Points show per species means of strategies scores. Contour lines show two-dimensional probability density distributions of strategies scores derived by kernel density estimations. The color gradient indicates regions of highest (dark) and lowest (white) occurrence probability of species with the given strategy.

**Figure 7:**
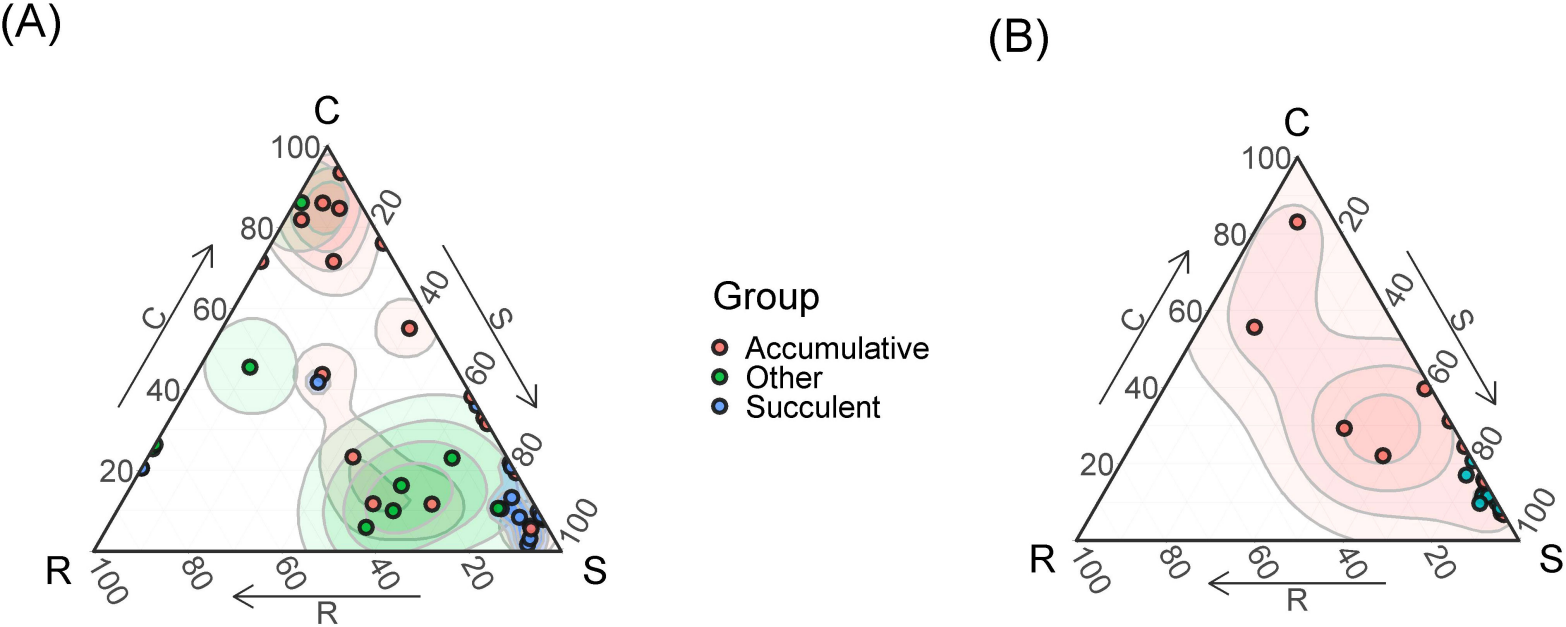
The SCR strategies of epiphytes belonging to different functional groups (accumulative epiphytes, succulents and others) in primary **(A)** and secondary **(B)** forests. Points show per species means of strategies scores. Contour lines show two-dimensional probability density distributions of strategies scores derived by kernel density estimations. The color gradient indicates regions of highest (dark) and lowest (white) occurrence probability of species with the given strategy.

The values of functional traits and strategy scores of epiphytes did not differ significantly between communities (**Figure 3**; **Supplementary Table**). In all the communities LDMC of epiphytes was significantly lower than in woody plants, and in LF, FP and LP communities it was significantly lower than in terrestrial herbs (**Figure 3F**). In contrast, size traits LDM and LA of epiphytes did not differ from terrestrial plants in all the communities (**Figures 3A, B**). In MHF epiphyte leaves were significantly thicker than in trees and lianas, and in LP they were thicker than in both woody and herbaceous terrestrial plants (**Figure 3C**). The SLA of epiphytes was lower than of terrestrial plants in LP, lower than in trees in MHF and lower than in herbs in FPC (**Figure 3D**). The LSI of epiphytes was higher than of trees in MHF and higher than of both trees and herbs in LP (**Figure 3E**). In the FPC and LP the stress-toleracy (S) score was higher in epiphytes than in terrestrial herbs, and the ruderality (R) score was lower (**Figures 3H, I**). Although mean values of epiphyte leaf traits and strategy scores do not differ significantly between forest types (**Figure 3**; **Supplementary Table**), the distribution of CSR strategies among species, and the taxonomic composition of epiphytes, change markedly between primary and secondary forests (**Figures 4**–**7**).

### 3.3 CSR strategies of epiphytes

Comparison of the CSR strategies of epiphytic and non-epiphytic plants (**Figure 4**) shows that woody plants are predominantly CS- strategy. The strategies of terrestrial herbaceous plants are diverse, but do not tend to the extreme points (**Figures 4B, D**). Epiphytes adhere to more polar values of strategies compared to terrestrial plants (**Figures 4A, C**). Most epiphytes tend to the stress-tolerant strategy. A smaller part of epiphytes is concentrated in the upper part of the triangle, i.e., adheres to the C-strategy. In general, epiphytes, compared to terrestrial plants, give more polar positions and tend to the vertices or edges of the triangle, while terrestrial plants are concentrated closer to the middle.

The taxonomic composition of epiphytes also has different trends in the nature of the distribution of CSR strategies (**Figure 5**). The diversity of primary forests is expectedly higher (**Figures 5A, C**). Orchids, which tend to the ruderal strategy (which is unexpected), drop out of secondary formations, as well as ferns and representatives of other taxonomic groups other than orchids and ferns, which tend to the competitive strategy (**Figures 5B, D**). In addition, orchids and ferns from the central part of the triangle, which are characterized by a mixed CSR-strategy, drop out.

Based on the comparison of epiphyte CSR strategies depending on their synusium affiliation (**Figure 6**), it can be concluded that stress-tolerant heliophytes are poorly represented in the primary forest (**Figure 6A**). In secondary communities, stress-tolerant sciophytes, mixed and competitive mid-stem species are poorly represented – at low altitudes they are replaced by stress-tolerant heliophytes (**Figure 6B**).

If we compare the CSR strategies of epiphytes taking into account their life forms (**Figure 7**), it is noticeable that the majority of stress-tolerant epiphytes are succulents, while accumulative epiphytes have various strategies: predominantly competitive (C), ruderal-stress-tolerant (SR) and mixed (CSR). Some accumulative and succulent epiphytes also adhere to the R-strategy. Other life forms are poorly represented.

## 4 Discussion

The collected dataset of morphological functional traits of the leaf consisted of 350 samples of epiphyte leaves belonging to 46 species. For comparison, in a meta-study of functional traits of epiphytes (Hietz et al., 2021), based on the global dataset, various morphological traits of the leaf were measured for 400–600 epiphytic species, out of about 2800 epiphytes in the dataset, and many epiphytic species were measured only once. In secondary formations, the variance of all traits is lower because fewer samples were collected due to the fact that there are few epiphytes in this formation. Specific leaf area differs significantly in all groups, which is expected: in disturbed formations, leaves are thicker, designating adaptation to water stress and which may be associated with a drier and hotter microclimate in the forest canopy. The obtained result is consistent with the results of Richards and Damschen (2022), who measured the functional traits of vascular epiphytes in northern Nicaragua on coffee plantations and in a tropical forest: SLA was statistically significantly higher in forests and lower in coffee plantations. SLA in undisturbed formations also differs among themselves. The leaf sizes of epiphytes within one formation can vary greatly - from about 1 cm^2^ in small-leaved species such as *Dendrobium oligophyllum* to about 0.25 m^2^ in *Asplenium nidus* and *Drynaria quercifolia*. In this study, the specific leaf area turned out to be more significant for epiphytes than individual morphological functional traits – leaf area, water-saturated leaf mass, air-dry leaf mass. The results here were expected: for “simple” morphological traits (LA, LDM, LFM), a small dispersion between the lower limitation of the sample and the median is characteristic, that is, in each sample there were about 50% of values – 50% of samples with small leaves, which is quite a lot. At the same time, each of the simple traits is characterized by the presence of outliers and, in principle, a high upper limit – this is due to the fact that each formation contains epiphytes, usually *Drynaria quercifolia, Asplenium nidus* or *Microsorum punctatum* with very large leaves. The area of the largest leaf counted, belonging to *Drynaria*, was 3661.81 cm^2^, and the smallest, which also belonged to one of the *Dendrobium* species, was 0.519 cm^2^. Moreover, each formation contained both species with large and small leaves. This leads to a large dispersion with relatively small median values of leaf mass, regardless of the water content in the leaf. Outliers in the diagrams are species with giant leaves. SLA, due to the fact that it is an integral indicator, makes it easier to detect differences in all groups. In primary formations, the specific leaf surface is greater than in secondary ones (**Figure 3D**). However, the most striking parameters that differed in epiphytes from terrestrial plants were LTH and LSI, the values of which increase in secondary habitats (**Figures 3C, E**). This correlates well with the tendency we discovered to strengthen the S-strategy when disturbed in epiphytes. Thus, our main disturbance signal is not a shift in average epiphyte trait values across forest types, but a shift, or mixture, of species and families occupying different regions of the CSR triangle, together with a simplification of taxonomic and layer structure (**Figures 4**-**7**). This is confirmed by our earlier works showing how the main groups of epiphytes Orchidaceae and Polypodiaceae react differently to factors of disturbance and simplification of the forest layer structure (Eskov, 2013; Eskov et al., 2020).

Traditionally, epiphytes are considered as stress-tolerant plants based on their morphological and chemical functional traits (Eskov et al., 2023; Eskov and Kolomeitseva, 2022; Zotz, 2016). As we expected, epiphytes would occupy the corner of radical stress tolerance in Grime diagrams with an increase in secondary habitats. However, we found that, unlike trees and terrestrial herbs, epiphytes gravitate toward more extreme manifestations of CSR strategies, and a number of epiphytes occupy the corners of the diagram with close to 100% competitive and even ruderal strategies. At the same time, tropical trees turned out to be closer to a mixed, competitive-stress-tolerant (CS) strategy, and terrestrial herbs to a mixed competitive-ruderal (CR) strategy. At the same time, it should be noted that, despite the unexpected results, there are still more epiphytic stress-tolerants than other epiphytes. In disturbed communities, epiphytes of ruderal, mixed and most competitive strategies disappear, and mainly stress-tolerant species remain. What could be the reason for the spread of unexpected CSR strategies of epiphytes in the lower tiers of a high-stemmed tropical forest? There are probably several reasons, and all of them are closely related to the characteristics of the lower and middle canopy of the forest as a habitat.

1. The distribution of the ruderal strategy seems the most uncommon, because ruderal species are those that easily recover from mechanical damage (Grime, 1988). In our case, these are mainly orchids belonging to different synusia, mainly succulents and sciophytes. It seems that there is not much mechanical damage in the crowns, however, the height of the trees of the first tier can reach 40–50 m; there are many mammals in the crowns (gibbons, macaques, langurs), which knock down epiphytes (Perry, 1978); bark, broken branches, less fortunate epiphytes and lianas fall from above. Branch litter makes up 10-30% of the mass of the entire litter (6–7 t/ha) (Kuznetsov, 2003). The impact of fallen deadwood on epiphytic communities has been noted by many researchers (Gomez-Escamilla et al., 2017; Hietz, 1997; Zotz, 1998; Zotz et al., 2005, 2021; Cabral et al., 2015). The ruderal strategy (as defined by J.P. Grime) is also followed by terrestrial annual plants, forced to survive the unfavorable period in the form of seeds. At altitudes of up to 5 m, the epiphytes noted by us were observed mainly on the bark of lagerstroemia and on thin branches of randia. In a tropical high-stem forest, there is one event that affects the seasonal availability of the substrate in the life of epiphytes: the change of bark in lagerstroemia. In addition, species living in the lower canopy of tall forests often live on thin branches of 3rd tier trees and therefore develop quickly in order to flower and produce fruits before the thin branches break under their weight (Chase et al., 2005). The influence of tropical monsoons is not so noticeable in Cat Tien (Deshcherevskaya et al., 2013), so it can be excluded.
2. The competitive strategy in the canopy of a high-stem tropical forest is predominantly followed by accumulative ferns living in the middle part of the trunk, such as *Asplenium nidus* and *Drynaria quercifolia*. Grime (1988) characterizes representatives of this strategy as having a high relative growth rate, a short leaf lifespan, and the ability to quickly capture resources through the spread of shoots and roots. Unexpectedly and unusually, a number of epiphytes turned out to be more pronounced competitors than trees! Despite the lack of a comparison of strategies, it was noted (Hietz et al., 2021) that epiphytes have to compete for light with other trees. In this case, rapid spread by creeping rhizomes allows the epiphyte to shade its neighbors and receive more light. In addition, developed roots allow the epiphyte to attach more firmly to the substrate and support more weight. The accumulation of suspended soils in “baskets” and “brackets” allows these epiphytes to obtain more mineral nutrients and moisture, allowing them to further increase their biomass (Abakumov and Eskov, 2023; Eskov et al., 2025). Finally, long-rhizome ferns can literally “run away” from the shading branches up the trunk, which is consistent with the assumptions and observations of other authors (Johansson, 1974; Guzmán-Jacob et al., 2022; Richards and Damschen, 2022). The tree trunk on which such epiphytes live is a fairly reliable and durable support, unlike thin branches; the only threat is a fall due to too much mass. Microclimatic conditions in a tropical high-trunk forest, in the middle part of the trunk, in particular air humidity, are quite stable. At the same time, during the dry season, the topsoil horizon dries out, the roots in it die, and the trees lose their leaves to reduce moisture consumption (Kuznetsov et al., 2010, Kuznetsov, 2003). Probably, adaptations to the dry season in trees shift them towards stress tolerance in the diagram (Peguero-Pina et al., 2020).
3. The distribution of stress-tolerant heliophyte orchids and ferns in disturbed communities was expected. The abundance of succulents suggests that the limiting factor is moisture availability, which is consistent with our hypothesis: it was assumed that due to lesser density, less shading (which is noticeable by the larger area occupied by herbaceous plants in the canopy of the secondary forest), and a smaller number of tree layers, the lack of moisture and daily fluctuations in air humidity are more pronounced here than in the undisturbed high-stem forest (Eskov, 2013; Eskov et al., 2020).

As expected, the proportions of heliophyte, mid-trunk and sciophyte species differ in disturbed and undisturbed formations: heliophyte species are most numerous in disturbed communities (68%), while sciophyte species are least numerous (8%). However, it is quite unexpected that mid-trunk epiphytes account for a larger proportion of the rest in undisturbed communities (50%). Presumably, the relatively small number of heliophyte species recorded in undisturbed communities is caused by difficulties in a field observation: epiphytic species living at the tops of trunks are not very visible with binoculars from the ground; in addition, as noted above, the inhabitants of the upper zones of trees tend to miniaturize (Perry, 1978; Chase, 1987; Johansson, 1974), making them difficult to detect from the ground even with binoculars. In our previous studies in this region (Eskov et al., 2020; Eskov, 2013), a strong dependence of the epiphytic community composition on the tier structure of the forest stand was noted: in disturbed communities, the synusia of sciophytic species was lost, the dominant Orchidaceae representatives in the undisturbed forest almost completely disappeared from the community and were replaced by ferns. However, in this study, we observe an abundance of epiphytic orchids in secondary habitats as well. Primary forests are diagnosed by the presence of the Zingiberaceae and Araceae families; in addition, the species composition of epiphytes between them and disturbed sites is fundamentally different. Is it legitimate to associate this with the course of the succession process? Rather no than yes, because we cannot speak about the full restoration of destroyed epiphytic vegetation in linear plantings, in the forest belt. The concept of succession in relation to epiphytic communities is itself a rather open question, since in the case of epiphyte colonization, we are not dealing with succession as a sequential change of species, but with “accumulation”, since phorophytes seem to accumulate epiphytes during their life (Flores-Palacios and García-Franco, 2006; Benavides et al., 2006; Zotz, 1999). It is important to note that in the forest belt and in cleared plantings, epiphytes were concentrated unevenly, most of the diversity and abundance of epiphytes were concentrated on relatively low, stocky “garbage trees” of the genus *Lagerstroemia*. It should be noted that for secondary forests with a single tree stand layer, the almost complete absence of epiphytic species and the low number of epiphytes are in themselves indicators of disturbances - because for primary formations of tropical mixed forests in South Vietnam, the presence of epiphytes is one of the defining ecological and physiognomic features (Kuznetsov, 2003; Eskov et al., 2020; Eskov, 2013).

Summarizing the abovementioned facts, it can be noted that secondary forests are characterized by the loss of synusia of sciophytic epiphytes and the depletion of synusia of mid-trunk ones, instead of them the lower tier of the forest is occupied by heliophytic succulent orchids and ferns. Heliophilic epiphytes seem to descend downwards in the secondary forest. The richness and abundance of epiphytes is higher in the primary forest, which has retained its tier structure. Epiphytes are characterized by a gravitation towards extreme variants of the CSR strategy, while less specialized species drop out of the community, while heliophytic succulents gain an advantage. The most interesting point is the fact that epiphytic species of the ruderal (P) strategy, according to the approach used in this work (Grime and Pierce, 2012; Pierce et al., 2017), are not diagnostic for forest disturbances. The abundance of stress-tolerant (S) epiphytes is the first indicator of forest disturbances.

## 5 Conclusion

In conclusion, our study can be summarized by the following findings and suggestions. (1) In the studied formations, 46 epiphytic species from 9 families were noted. In the primary formations, representatives of the Orchidaceae family dominate in terms of species, while in the disturbed ones, Orchidaceae and Polypodiaceae dominate. (2) The values ​​of functional traits of epiphytes did not differ significantly between communities. Epiphytes tend to have a more pronounced C-strategy than tropical forest trees and a more R-strategy than terrestrial herbs. At the same time, most epiphytes tend to have a radical S-strategy. (3) Differences were noted between the CSR strategies of epiphytic communities living in primary and secondary forests: in the former, epiphytes adhering to C, R, and mixed strategies are more widely represented. Representatives of these strategies disappear in secondary forests so that mainly stress-tolerant species remain. Thus, our hypothesis is confirmed. (4) In the studied secondary formations of tropical forest, the lower forest layer is occupied mainly by stress-tolerant succulent orchids and ferns. The primary mature tropical forest is distinguished by the presence of sciophyte epiphytes and a richer synusium of mid-trunk species. Disruption of the structure of the tropical forest leads to the loss of epiphytic species of the lower synusia, while the advantage goes to stress-tolerant heliophyte succulents.

Thus, main disturbance signal is not a shift in average epiphyte trait values across forest types, but a shift, or mixture, of species and families occupying different regions of the CSR triangle, together with a simplification of taxonomic and layer structure. From the above, it can be concluded that the response of epiphytes to disturbances is largely explained by a phylogenetic signal, which requires further study.

## 6 Limitations of the study and its prospects

The main limiting factor of this study was the difficulty in collecting epiphyte samples, as mentioned above. With current collection methods, it is impossible to collect all epiphytes from a tree without felling it. In tall forests, collection using hooks on poles systematically undercounts small heliophyte epiphytes, especially in the peripheral areas of the canopy. This somewhat distorts our data, especially for tall forests. However, we previously conducted research in this forest using industrial mountaineering methods (where a researcher climbed trees and collected epiphytes manually) (Eskov et al., 2019, 2020), and we can conclude that the taxonomic picture of the epiphyte communities studied previously and currently does not differ significantly. It’s also important to consider our data in the context of the limitations of the method used. Epiphytes have fundamentally different constraints (lack of root access to soil, uptake of atmospheric water and nutrients, and mechanical anchorage requirements, which may invalidate the relationships between traits and stress tolerance). The method uses only three leaf traits (LSI, LDMC, SLA), which may not reflect epiphyte-specific adaptations such as pseudobulbs, water storage reservoirs, or specialized root structures. We can view this study as a pilot for epiphytes, providing a good opportunity to initiate discussions about the development (or adaptation) of global, simple, and universal methods for studying functional ecology for all vascular plants. Epiphytes contribute disproportionately to most global centers of plant diversity and play an important role in creating the global gradient of latitudinal plant diversity (Taylor et al., 2021), and constitute approximately 10% of all vascular plant species, making their inclusion in any plant functional diversity research project inevitable.

## Data Availability

The raw data supporting the conclusions of this article will be made available by the authors, without undue reservation.
