# Supplementary material for: Tropical forest disturbances reveal increase in stress-tolerant(s) strategy among epiphytes while simplifying the taxonomic and layer structure of epiphytic communities
Source: Front Plant Sci. 2026 Feb 18;17:1695534. doi: 10.3389/fpls.2026.1695534 (PMC12957178; doi:10.3389/fpls.2026.1695534)
Supplement: Supplementary file 1 [file Table1.docx]

**Supplementary Table.** The results of approximative k-sample Fisher-Pitman permutation test and pairwise comparisons of leaf traits ans strategies scores between groups (epiphytes, trees and terrestrial herbs) within communities. p-values of pairwise comparisons are given with false discovery rate corrections. Only significant values are included. SLA – specific leaf area. LSI – leaf succulence index. LWC – leaf water content. LDMC – leaf dry matter content. LMA – leaf mass per area. Strategies: C – competitors, S – stress-tolerators, R – ruderals. Communities: MHF – mixed high forest, LF – *Lagerstroemia* forest, CLF – crooked low forest, FP – forest planting, FPC – forest planting clear, LP – linear plantings.

| Community | Plant traits | χ^2^ | p | Pairwise comparisons | p (FDR-corrected) |
| --- | --- | --- | --- | --- | --- |
| MHF | S strategy | 6.0605 | 0.0438 | Herbs – Trees | 0.016 |
|  | R strategy | 7.9389 | 0.0166 | Herbs – Trees | 0.012 |
|  |  |  |  | Epiphytes – Herbs | 0.035 |
|  | Leaf thickness | 7.1478 | 0.0165 | Epiphytes – Trees | 0.044 |
|  | SLA | 15.279 | <0.001 | Herbs – Trees | 0.008 |
|  |  |  |  | Epiphytes – Herbs | 0.007 |
|  | LSI | 8.5705 | 0.112 | Epiphytes – Trees | 0.022 |
|  | LWC, LDMC | 13.9 | <0.001 | Herbs – Trees | 0.022 |
|  |  |  |  | Epiphytes – Trees | 0.001 |
| LF | LWC, LDMC | 9.611 | 0.003 | Epiphytes – Trees | 0.015 |
|  |  |  |  | Epiphytes – Herbs | 0.015 |
| CLF | LWC, LDMC | 9.241 | <0.001 | Epiphytes – Trees | 0.011 |
| FP | S strategy | 6.921 | 0.018 | Epiphytes – Trees | 0.070 |
|  |  |  |  | Epiphytes – Herbs | 0.070 |
|  | LWC, LDMC | 6.710 | 0.003 | Epiphytes – Trees | 0.072 |
|  |  |  |  | Epiphytes – Herbs | 0.072 |
| FPC | S strategy | 6.177 | 0.035 | Epiphytes – Herbs | 0.050 |
|  |  |  |  | Herbs – Trees | 0.050 |
|  | R strategy | 9.352 | 0.006 | Epiphytes – Herbs | 0.024 |
|  | SLA | 9.96 | 0.005 | Epiphytes – Herbs | 0.019 |
|  | LWC, LDMC | 11.709 | <0.001 | Epiphytes – Trees | 0.006 |
|  |  |  |  | Herbs – Trees | 0.025 |
|  | LMA | 6.506 | 0.027 | Epiphytes – Herbs | 0.071 |
| LP | S strategy | 19.252 | <0.001 | Epiphytes – Herbs | <0.001 |
|  |  |  |  | Epiphytes – Trees | 0.001 |
|  | C strategy | 9.593 | 0.005 | Epiphytes – Trees | 0.022 |
|  |  |  |  | Herbs – Trees | 0.040 |
|  | R strategy | 18.686 | <0.001 | Epiphytes – Herbs | <0.001 |
|  |  |  |  | Epiphytes – Trees | 0.020 |
|  |  |  |  | Herbs – Trees | 0.024 |
|  | Leaf thickness | 25.111 | <0.001 | Epiphytes – Herbs | <0.001 |
|  |  |  |  | Epiphytes – Trees | <0.001 |
|  |  |  |  | Herbs – Trees | 0.049 |
|  | SLA | 18.079 | <0.001 | Epiphytes – Herbs | <0.001 |
|  |  |  |  | Epiphytes – Trees | 0.006 |
|  | LSI | 24.613 | <0.001 | Epiphytes – Herbs | <0.001 |
|  |  |  |  | Epiphytes – Trees | <0.001 |
|  | LWC, LDMC | 18.4 | <0.001 | Epiphytes – Herbs | <0.001 |
|  |  |  |  | Epiphytes – Trees | <0.001 |
|  | LMA | 18.859 | <0.001 | Epiphytes – Herbs | <0.001 |
|  |  |  |  | Epiphytes – Trees | <0.001 |
|  |  |  |  | Herbs – Trees | 0.035 |
